# The application of discrete choice experiments eliciting young peoples’ preferences for healthcare: a systematic literature review

**DOI:** 10.1007/s10198-022-01528-9

**Published:** 2022-09-28

**Authors:** Galina Williams, Irina Kinchin

**Affiliations:** 1 School of Business, Accounting and Law, CQUniversity, Brisbane, Australia; 2 Centre for Health Policy and Management, School of Medicine, Trinity College Dublin, The University of Dublin, Dublin, Ireland

**Keywords:** Stated preferences, Discrete choice experiment, Youth, Adolescents, Health services, Health policy, I12, I18, C93, C52, D12

## Abstract

**Objectives:**

Understanding young people’s preferences for healthcare is critical for reducing the negative effect of undesirable choices. This review aims to synthesise the evidence obtained from discrete choice experiments (DCEs) eliciting young people’s preferences for healthcare interventions and service deliveries, specifically, to (1) examine the methodology, including a selection of attributes and levels, experimental design, estimation procedure and validity; (2) evaluate similarities, differences and rigour of designs to the general population DCEs; and, (3) compare the DCEs’ application to the seven health priority areas defined by the World Health Organisation (WHO).

**Methods:**

A systematic review searching Medline, EconLIT, PsychINFO, Scopus, and Web of Science was performed up until May 2021. Inclusion criteria: a DCE, eliciting young peoples’ preferences (10–24 years of age), on a healthcare-related topic defined by WHO, peer-reviewed, full-text available in English. A bespoke checklist was used to assess the methodological quality of the included studies.

**Results:**

Eighteen DCE studies were included in the review, exploring interventions and service in sexual and reproductive health (*n* = 9; 50%), smoking cessation (*n* = 4; 22%), mental health (*n* = 1), nutrition (*n* = 1), unintentional injuries (*n* = 1), vaccination against severe but rare diseases (*n* = 1); and diabetes (*n* = 1). Compared to the general population, DCEs eliciting young people’s preferences had a high proportion of monetary measures and a smaller number of choices per respondent with the overwhelming number of surveys using fractional factorial design. The majority of studies were of moderate quality (50–75% of the criteria met).

**Conclusions:**

While identified DCEs touched on most health priority areas, the scope was limited. The conduct and reporting of DCEs with young people could be improved by including the state-of-the-art design, estimation procedures and analysis.

**Supplementary Information:**

The online version contains supplementary material available at 10.1007/s10198-022-01528-9.

## Introduction

Discrete choice experiments (DCEs) within health economics have grown in popularity in recent years [1]. Increasing demand for healthcare warrants efficient allocation of scarce resources available to healthcare. Non-market valuation techniques such as DCEs can assist in informing policymakers regarding demand preferences and trade-offs that individuals are willing to make for different attributes of healthcare as well as the level of expected demand [2]. In a DCE, respondents repeatedly choose between two or more alternatives to reveal a latent utility function. They trade off risks and benefits among alternative scenarios and express their choice by selecting a preferred option. This technique draws on elements of random utility theory, consumer theory, and experimental design theory [3]. Data from DCEs can be used to estimate relative values of different attributes and their levels and willingness to pay or willingness to accept specific healthcare interventions and service deliveries [2]. Details on theory and how to conduct DCEs are covered elsewhere [3, 4].

Young people is a critical cohort, particularly concerning healthcare delivery. Young people's choices can lead to profound and long-lasting implications for employment, wealth, and health [5]. This age group has higher mortality rates from land transport accidents, unintentional injuries, mental health, violence, perinatal, and congenital conditions than those of the general population [6, 7]. According to the World Health Organisation (WHO) [7] more than 2 million adolescents were living with HIV (primarily in the African region). Although HIV-related deaths have decreased overall, but not among adolescents [7]. The unhealthy patterns of risk behaviour at a young age, such as smoking, risky substance use, obesity, and sexually transmitted diseases, affect young people's health and can have a delayed effect on adult health and might present future costs [5, 8]. Early interventions, which can be more effective in adolescence than in adulthood, can prevent delayed harm [9]. Further evidence suggests that young people form explicit opinions about healthcare service delivery [10, 11]. Researching their preferences is vital for understanding determinants of healthcare demand and designing effective health promotion and early intervention strategies.

Previous systematic reviews concentrated on the application of DCEs with the general population [12], on trends [13–15], and on characteristics of DCEs [14, 15]. A few reviews focused on specific health areas, such as nephrology [13], concordance and discordance of patient and health care provider [16], and the types of programs that are more likely to be taken up given their characteristics [17]. To date, no study has examined the use of DCEs with young people.

This study systematically reviewed the application of DCEs eliciting young peoples' preferences (as respondents) for healthcare interventions and service deliveries. Using the WHO [7] terminology, adolescents were defined as 10–19 years old, youth as 15–24 years old, and young people as between 10 and 24 years of age. Specifically, this review aimed to (1) examine the methodology, including a selection of attributes and levels, experimental design, preference measurement, estimation procedure, and validity; (2) evaluate similarities, differences, and rigour of designs and compare these to DCEs with the general population; and, (3) compare the DCEs application with the health priority areas defined by WHO. WHO defines health priority areas globally, which were used to classify health areas of studies included in this review. The rest of the paper is structured as follows. The next section presents methods. The subsequent section provides the results. The final section discusses the findings and identifies areas for future research.

## Methods

### Protocol and registration

The authors followed an a priori protocol registered with PROSPERO (CRD 42017082161). The review was prepared under the Preferred Reporting Items for Systematic reviews and Meta-Analyses (PRISMA) guidelines [18].

### Information sources and search strategy

The following five electronic databases were searched to achieve comprehensive coverage of the global healthcare and economics literature focusing on youth: Medline, EconLit, PsychINFO, Scopus, and Web of Science. The initial search was conducted in February 2019 and was later updated in May 2021. The search was conducted separately for each database and used database-specific search strings.

The search strategy combined keywords and MeSH terms in either title, abstract or MESH heading generally describing and pertaining to three main groups including discrete choice experiments, youth and health.

1. **Discrete choice:** choice model* OR choice experiment OR choice behav* OR part-worth utilities OR discrete choice OR best*worst scaling OR functional measurement OR paired comparisons OR pairwise choice* OR stated preference
2. **Youth:** adolescent* OR youth* OR young people OR young adult* OR child* OR teenage* OR juvenile*
3. **Health:** health OR wellbeing OR empowerment OR psychosocial OR growth OR development OR self-efficacy OR self-confidence OR coping OR mortality OR morbidity OR stress OR anxiety OR depression OR harm* OR access OR satisfaction OR health knowledge OR attitudes OR behaviour OR practice OR understanding OR health seeking OR quality of health care OR quality of life
4. **Exclusions**: NOT parent* NOT education NOT crime NOT friendship

The final search was defined as the combinations of groups of terms: discrete choice AND youth AND health. Only studies published in English were included. Backwards snowballing or reviewing references supplemented the database searching. The search was validated by checking all relevant references from other systematic reviews [13–15].

### Study selection

After the removal of duplicates, the titles and abstracts of retained references were screened for relevance. Both authors independently reviewed the list. Studies were included if they were experimental, as opposed to solely regarding methodology or theory, and if they included analysis of choice-based response data, as opposed to rating or ranking exercises; focused on a healthcare-related topic or condition; elicited youth preferences using a DCE methodology; were peer-reviewed with a full text available. A youth group was defined as a population aged 10–24 years according to the WHO range for adolescents, youth and young people. Studies that included other age groups or parents/caregivers choices were excluded, as well as studies that did not use Random Utility Theory, such as adapted conjoint analysis. Studies in which more than two-thirds of respondents were 10–24 years old (under 30 years old) were included. This review adopted the health priority taxonomy by WHO [24]. Studies with the areas of application outside of this taxonomy were excluded.

After independent title and abstract review, there was a 90% agreement between the authors, who then met to resolve any disagreement by discussion. If a study could not be excluded with certainty, it was included in the full-text review. Ambiguous cases were resolved by discussion.

### Data collection and quality assessment

Data extraction focused on describing the main stages of DCEs identified from a checklist for critical appraisal and based on the choice task design, analysis and interpretation [2]. These data included methods for designing choices and attributes used in the DCE, piloting, study samples, framing, marginal rates of substitution and the analysis, including consideration of subgroups and heterogeneity. Detailed information was extracted from each study and included the lead author and publication year, country, health area (disease), intervention, samples compared, attributes covered, design type, plan and source, methods to create choice sets, piloting of studies, framing of choice tasks, number of attributes, source of attributes and level assignment, priors, number of choices per respondent, administration of the survey, response rate, completion time, D-efficiency, econometric models, validity and reliability. Further details are provided in a supplementary file.

The quality of DCE studies can be assessed using various matrices [19–22]. We designed a bespoke checklist following the methodology by de Bekker-Grob et al. [15], Lancsar and Louviere [2], and Louviere and Lancsar [23] [19–22]. The quality criteria covered several stages of DCEs, such as design of choice tasks, piloting and results consistency, as poor design or conduct of the DCE cannot be overcome in the analysis [22]. We assessed whether each criterion for each study was met or not. If there was insufficient information to judge the quality of a criterion, we noted this as a separate category termed “not met or not explicitly mentioned”.

Results were compared with de Bekker-Grob et al. [15] and Soekhai et al. [14]^1^ to identify differences and gaps in the DCEs with young people using ratios. Ratios to Bekker-Grob et al. [14] and Soekhai et al. [15] were calculated by using the percentage of studies in the current review to the percentage of studies in the corresponding reviews by Bekker-Grob et al. [14] and Soekhai et al. [15] accordingly.

### Synthesis of results

The WHO prioritises several health areas with immediate health risk consequences, affecting healthy, productive adulthood and future generations globally. These areas were used to classify DCEs included in this review [24]. The areas identified by WHO [24] were:

- Target area 1: Unintentional injuries and violence.
  Unintended injuries and violence affect young people more than other age groups and account for about 9% of disability-adjusted life years in the young people group.
- Target area 2: Mental and neurological health the high burden from these conditions on young people.
- Target area 3: Sexual and reproductive health, including HIV infection and HIV-related illnesses
  HIV/AIDS is one of the top five causes of death in this age group. It stated that the impact of teenage pregnancy includes intergenerational effect on newborns who have a higher risk of dying than infants born to older mothers.
- Target area 4: Nutrition an inadequate nutrition in childhood affects the growth and development potential of young people and the pregnancy of adolescent girls, including the risk of infant's developing obesity and other diseases later in life.
- Target area 5: Alcohol and illicit drug use alcohol is the largest contributor to risks to health in young people. It is associated with the risks for the unborn child, increased injury risk, violence, mental health issues and unsafe sex, including HIV infections. Illicit drug use is the second largest contributor to risks to health in young people.
- Target area 6: Other behaviours behaviours formed during adolescence could continue in adulthood, affecting health. About 17% of the world's burden of disease in all ages is attributed to unprotected sex, tobacco smoking, alcohol, and illicit drugs consumption as well as physical inactivity.

## Results

### Description of studies

**Fig. 1:**
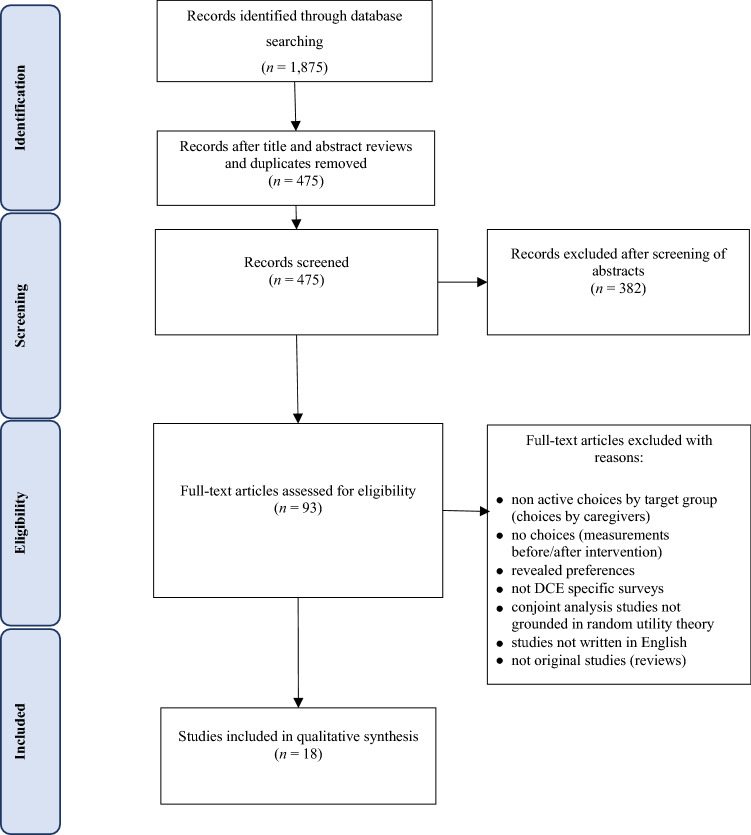
PRISMA flowchart

The search strategy resulted in 1,875 hits, from which 475 studies were retrieved after the title and abstract review. After a full-text review, 18 papers met the inclusion criteria (Fig. 1). Given the limited and heterogeneous nature of included studies, a descriptive analysis is presented below.

A large proportion of included studies originated from Sub-Saharan Africa (*n* = 6; South Africa, Malawi and Zimbabwe), followed by North America (*n* = 4; Canada and USA) and Europe (*n* = 4; including Netherlands, Switzerland, and Ireland), Eastern Mediterranean Region (*n* = 2, Lebanon and one study covered Palestine, Jordan, UAE and Oman) and Asia/Pacific (*n* = 1, Australia) (Table 1). One study involved two countries within the same study setting [25]. The majority of studies were published between 2015 and 2021 (*n* = 8; 44%) (Table 2). More than 60% of the studies (*n* = 11) came from high-income countries, in contrast to 89% of studies that were reported in the most recent review of DCEs with the general population [15] (Table 1).

**Table 1:** Comparison of DCEs eliciting young people’s preferences versus DCEs in general

Characteristic	Number of studies in current review (*n*; % of total)	Percentage of de Bekker-Grob et al. [15]	Percentage of Soekhai et al. [14]
High-income country (GNI per capita of US$12,236 or more)	11 (61%)	69%	89%
Regions			
Europe	4 (22%)	37%	60%
North America	5 (28%)	158%	122%
Sub-Saharan Africa	6 (33%)	NA	NA
Eastern Mediterranean Region	2 (11%)	NA	NA
Asia/Pacific	1 (6%)	49%	61%

**Table 2:** Overview of DCEs eliciting young people’s preferences

Characteristic	Number of studies in current review (*n*; % of total)
Year (published)	
< 2010	0 (0%)
2010–2014	4 (22%)
2015–2021	14 (78%)
Health area	
Mental health	1 (6%)
Nutrition	1 (6%)
Sexual and reproductive health	9 (50%)
Smoking	4 (22%)
Unintentional injuries (driving and alcohol)	1 (6%)
Vaccination (against severe but rare diseases)	1 (6%)
Diabetes	1 (6%)

### Areas of application

Table 2 provides a taxonomy of the DCEs application. The majority of identified DCEs examined sexual or reproductive health choices (*n* = 9 out of 18 studies; 50%), including three studies on preferences associated with HPV vaccination and three studies related to HIV (Table 2). A descriptive analysis of included studies is presented below. More details are provided in the Supplementary material.

### Target area 1: unintentional injuries and target area 5: alcohol and illicit drug use

An analysis by Scagnolari et al. of the mobility preferences of young people at nighttime revealed that driving a car was a preferred alternative to taking public transport [26]. Among those respondents who favoured using a car, the probability of being stopped by the police attribute was ranked first, with the amount of fine being the second-ranked attribute. For those who favoured using public transport, the level of service was ranked higher than the fare.

### Target area 2: mental health

One study assessed preferences for providing information about mood and anxiety disorders among young people in Canada [27]. This study identified three latent classes of respondents with different information preferences: those who preferred virtual information, conventional information and the low-interest classes. Both virtual and conventional information classes ranked the information content first, followed by the acquisition process. The low-interest class had the highest priority for the acquisition process variables. The source of supporting evidence was ranked highest among the virtual information and conventional information classes. In contrast, the low-interest class prioritised the time demand over the source of supporting evidence. Time demand was ranked second in the virtual information class, while the advertising channel held the second rank in the conventional information class.

### Target area 3: sexual and reproductive health

Nine studies examined preferences of young people for the provision of sexual or reproductive health care initiatives, and their contributing factors, including preferences for human papillomavirus (HPV) vaccination (*n* = 3); HIV (*n* = 4); integrated service delivery for family planning (FP) and HIV (*n* = 1); and specialised FP services delivery (*n* = 1) [11, 25, 28–34]. Three studies from high-income countries examined preferences associated with HPV vaccination, while six studies examined preferences for HIV and family planning testing and services in Malawi, Zimbabwe and South Africa.

The same attributes were applied in two studies from the Netherlands with the gap of three years, while the USA study used similar attributes. The results showed that the highest rank was for the vaccine with increasing cervical cancer protection attribute, followed by the duration of the protection and protection from genital warts [28]. Results of mixed logit analysis from the DCE study from the Netherlands revealed the following order of ranking (starting from the highest): serious side-effects, duration of protection and the effectiveness of protection against cervical cancer [29]. The lowest-ranked attributes were the mild side effects and the age of vaccination. A follow-up study from the Netherlands identified three latent classes of respondents [30]. The respondents from two latent classes ranked the degree of protection first, duration of protection second, and the mild side-effects third based on overall coefficients. The third latent class ranked the attributes in the following descending order: mild side effects, the duration of protection and degree of protection.

Studies on preferences for HIV self-testing in low-income countries, Malawi and Zimbabwe, were conducted using similar core attributes: price, location and pre-test support. The rest of attributes reflected differences between delivery of HIV testing in two countries. The results revealed the following ranking: the highest ranked attribute was the home location of self-test distribution. In Malawi, the highest ranked attribute was followed by the post-test support attribute and the type of provider. In Zimbabwe, other attributes were not statistically significant [25]. In South Africa, the preferences for HIV testing were elicited focusing on incentives attributes. The findings exhibited the following order of relative importance: support for HIV testing, location, and source of HIV information [31]. The preferences for HIV prevention were examined in [32] using similar attributes. The highest ranked attribute was dosage (1 year as a frequency of the procedure), followed by product form (as an injection as opposite to implant), and soreness (mild). Preferences of South African youth living with HIV were according to the following order of relative importance: program inclusion of participants, cash payments and clinic delivery [33].

The results from a study on preferences for family planning services revealed that in government (free) clinics the highest-ranked attribute was the availability of family planning commodities followed by the service provider attitude and distance to travel to the clinic [11]. The ranking of attributes was similar for the private clinic and the community-based distribution agent. However, waiting time at the facility was ranked third for the private clinic. The preferences for the outreach service had only one statistically significant variable apart from the cost, the waiting time [11]. A DCE from Malawi ranked the following attributes when designing a package of sexual and reproductive health and HIV services in Malawi: confidentiality of service, availability of tests and recreation and sports activities offered for youth [34].

The results of the DCE evaluating adolescent vaccination preferences revealed the following top ranking among six attributes. The highest WTP was for the life-threatening illness, followed by the delivery method and the way it was administered [35].

### Target area 4: nutrition

Two studies from high-income countries examined the role of branding and price in motivating healthier snack consumption [10, 36]. According to a study by Hartmann et al. children preferred product type over the brand, with the price being the less relevant to their choices. Another DCE from Ireland found that young adults preferred to see health professional(s) (i.e. nurse and consultant), type of glucose diary and availability of the optional services (i.e. dietitian and psychologist) [10]. That was closely followed by how the diabetes was tested and the choice of healthcare practitioner selected by the respondent attributes.

### Target area 6: smoking

Four studies covering both high- and low-income countries analysed the effect of cigarettes packaging on smoking (high-income country), preferences for cigarettes and e-cigarettes (high-income country), smoking cessation behaviours (low-income country), and waterpipe smoking (low-income country) [37–40]. Health warning attribute was included in some form in all of these studies. Studies found that respondents were willing to pay more to reduce the risk of smoking side effects than for the available support and distance travelled to stop smoking [37]. The pack structure (i.e. lipstick, slim, booklet or traditional) had the highest rank in the cigarette's choices, followed by being branded or plain. Lastly, the price had the lowest rank among significant coefficients [38]. When choosing between cigarette and e-cigarettes, the highest effect on the ranking was the harm of second-hand smoke, followed by flavour and whether it caused trouble breathing in the basic MNL model. Among those respondents who preferred smoking cigarettes, whether the cigarettes cause trouble breathing attribute had a second rank after the flavour. Vaping group' ranking was flavour, harm from second-hand smoke and causes trouble breathing attribute on the third rank [39]. Flavour was ranked first before the nicotine content in another study looking at the waterpipe smoking demand in the Eastern Mediterranean region [40].

### Attributes, choices and alternatives

Interviews and focus groups were the most used methods for identifying attributes and their levels. About 50% of studies (*n* = 9) used both methods, while about 83% (*n* = 16) used either an interview or a focus group. The following attributes were reported: monetary measures (*n* = 16, 89%), services (*n* = 16, 89%), risks (*n *= 8, 44%), time (*n* = 6, 33%) and the expected outcome (*n* = 4, 22%). There were fewer selected attributes in DCEs with young people than DCEs with the general population (Table 3). The number of attributes ranged from three to fifteen, with the most studies (*n* = 7, 44%) employing four to five attributes. This finding is consistent with the other DCE studies [14, 15] (Table 3).

**Table 3:** Attribute selection and level setting

Item	Category	N of studies in current review	%	Percentage of de Bekker-Grob et al. [15]	Percentage of Soekhai et al. [14]
Source of attributes and	Interviews, expert opinions	8	44%	NCR	NCR
level assignment	Focus groups	7	39%	NCR	NCR
	Interviews and focus groups	9	50%	NCR	NCR
	Pilot	11	61%	NCR	NCR
Number of attributes	2–3	2	11%	0.80	1.03
	4–5	9	47%	1.08	1.19
	6	5	26%	1.00	1.15
	7–9	2	11%	0.80	0.49
	10+	1	5%	1.50	0.96
Attributes covered	Monetary measure	16	89%	1.66	1.78
(by study)	Time	6	33%	0.66	0.86
	Risk	8	44%	1.45	1.01
	Expected outcome	4	22%	0.41	0.94
	Services	16	89%	1.28	2.57
	Others	9	50%	3.35	1.05
Number of choices per	4 or less	12	67%	NCR	NCR
respondent	5–8	3	17%	0.42	0.58
	9–16	3	17%	0.44	0.31
	> 16	1	6%	0.30	0.38
Administration of survey	Online	4	22%	NCR	0.78
	Computer/laptop	2	22%	3.90	NCR
	Paper	9	56%	0.83	2.42
Response rate (non-reported)		8	44%	NCR	NCR
Completion time (non-reported)		11	61%	NCR	NCR

*NCR* not clearly reported

The majority of studies included four or fewer choices per respondent. Most studies were administered using pencil and paper (*n* = 9, 56%). The rest of the studies used a computer or online method for survey administration. This finding contrasts with the DCEs with the general population, where most studies included nine to 16 choices per respondent, with online being the prevailing method of administration (57%) [14] (Table 3).

### Experimental design

A fractional factorial was the most frequently used design in DCEs with young people and the general population (Table 4). DCEs focused on young people's preferences tend to estimate the main effect. About 56% of studies did not report their design plan compared to 49% DCEs with the general population. DCEs focused on young people’s preferences primarily used Ngene (50%) followed by STATA, SPSS and SAS package (17%) to create an experimental design. All of those software packages can provide D-efficient designs. This was at odds with the general population DCEs, where the predominant choice of creating an experimental design remained SAS (25%). DCEs with young people reported the D-efficient design (72%) followed by the random allocation of profiles (22%). DCEs with the general population also used the D-efficient design (43%) and, to a lesser extent, the foldover design (9%).

**Table 4:** Experimental design and construction of choice sets

Item	Category	N of studies in current review	%	Percentage of de Bekker-Grob et al. [15]	Percentage of Soekhai et al. [14]
Design type	Full factorial	0	0%	NA	0
	Fractional factorial	16	89%	86.67	0.97
	NCR	2	14%	NCR	2.11
Design plan	Main effects only	3	17%	0.19	0.58
	Main effects, two-way interactions	5	28%	5.27	1.61
	NCR	10	56%	7.92	2.43
Design source	Software packages, Ngene	9	50%	NCR	2.43
	Software packages, STATA/SAS/SPSS	3	17%	0.68	0.68
	Other	2	11%	0.29	4.79
	NCR	4	22%	0.60	0.68
Methods to create choice sets	Binary	0	0%	0.00	0.00
	Random	4	22%	1.33	5.57
	foldover	1	6%	0.53	0.60
	D-efficiency	13	72%	5.88	1.70
	other	0	0%	0.00	0.00
	NCR	0	0%	0.00	0.00
Priors	Zero	3	11%	NCR	NCR
	Non-zero	5	28%	NCR	NCR
	NCR	11	61%	NCR	NCR

*NCR* not clearly reported

### Econometric analysis method

None of the studies used probit or random-effects probit. This finding supports the overall declining trend in the use of logit, probit and random effects probit reported by [13]. The increased use of mixed logit (MXL) analysis is evident in DCEs focused on young people’s preferences: about 40% of studies used MXL to analyse the respondents’ decisions. Neither DCEs focused on young people’s preferences, nor DCEs with the general population used nested logit. However, Latent Class 28% (13%) models that allow for preference heterogeneity were used more often in DCEs with young people than DCEs with the general population.

### Validity check

DCEs focused on young people’s preferences less frequently conducted validity and reliability tests compared to the DCEs with the general population [14] (50% and 58%, respectively) (Table 5). The outcome measures in the DCEs focused on young people’s preferences were centred around WTP 72% (50%), time 33% (39%) and risk 28% (44%) compared to DCEs with the general population, respectively. DCEs with the general population also used utility scores, odds ratios and probabilities as the primary outcome measure in addition to WTP [14].

**Table 5:** Estimation procedure and validity

Item	Category	N of studies in current review	%	Percentage of de Bekker-Grob et al. [15]	Percentage of Soekhai et al. [14]
Econometric models	Conditional Logit	3	17%	1.46	NA
	Random effects Logit	5	28%	5.28	5.57
	MLN	5	28%	1.27	0.60
	Nested Logit (NL)	0	0%	0.00	0.00
	Mixed Logit (MXL)	7	39%	7.39	1.00
	Latent Class (LCM)	5	28%	31.67	2.20
	Other	1	6%	0.11	0.52
	NCR	0	0%	0.00	0.00
Validity and reliability	Conducted	9	50%	0.72	0.86
	NCR	9	50%	NCR	1.19

*NCR* not clearly reported

### Quality assessment

The average quality score was 68% out of 100% (Table 6). There were no low-quality studies (< 50% of criteria met), with eight studies (44% of total studies) rated as high quality (> 75% of the criteria completed) and the rest as moderate quality (50–75% of the criteria met).

**Table 6:**
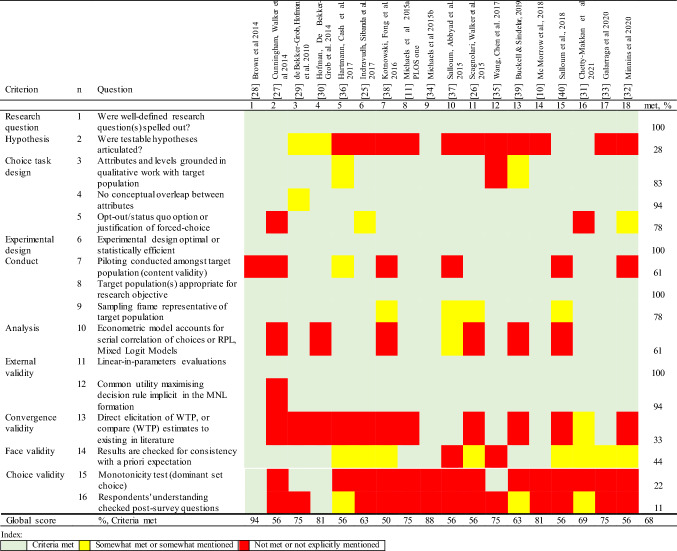
Quality assessment of included studies (*n* = 18)

All selected studies had a well-defined research question that specified clearly what the studies aimed to measure [19]. All studies reported their choice of experimental design with an appropriate target population and appropriate evaluation of the parameters. The majority of studies (89%) did not explicitly report the choice validity, for example, a formal check of the respondents' understanding of DCE questions and a monotonicity test. WTP/WTA was estimated only in 33% of all studies. Surprisingly, WTP estimates were not reported in all studies that used price as an attribute. About 60% reported results beyond MNL, such as random parameters logit and mixed logit. The majority of studies (60%) conducted a pilot study. About 78% included the opt-out status quo option or justified the forced choice. About half of the studies (44%) checked the results for consistency with prior expectations.

## Discussion

Adolescence and young adulthood’s behavioural risks and protective factors could affect current and future health and wellbeing. Therefore, understanding young people’s preferences for healthcare interventions and service deliveries is critical for reducing the negative effect of undesirable choices. This systematic literature review identified a growing number of DCEs eliciting young people's preferences for healthcare. This was the first review of the DCEs focusing on young people’s choices the best of our knowledge.

The majority of included studies came from high-income countries, with a third of studies being from Sub-Saharan Africa. All six health risk areas listed by WHO [24] as being of immediate consequences were identified by the review. Sexual and reproductive health, and smoking were the most studied areas. Most studies sources attributes by conducting interviews and working with focus groups and performing pilot studies. Almost 60% of included studies used no more than five attributes in order to reduce the cognitive burden on respondents.

While the use of DCEs in the healthcare context is growing [13, 14], this review identified several deficiencies in the DCEs application with young people. The conduct and reporting could be improved by including recent defelopment in DCE design, estimation procedures and analysis. Compared to the general population, DCEs eliciting young people's preferences had a high proportion of monetary measures and a smaller number of choices per respondent with the overwhelming number of surveys using fractional factorial design. While identified DCEs touched on most health priority areas, the scope was limited. Greater attention is warranted for such health areas as injury prevention and control, nutrition, access to mental health services, asthma and diabetes.

This review adapted a comprehensive search strategy and rigorous quality check. It identified trends specific to youth healthcare and compared those to the general population. This study included aspects of DCEs not investigated in the general population, such as specific health areas of application, source of attributes, response rate, completion time, and priors in the experimental design. The DCE literature recognises all these aspects as essential for conducting quality research [24].

Several limitations are also worth discussing. Although the protocol guided the rigorous and thorough search strategy, potential limitations include the possibility that the search did not locate all the relevant studies. This review took a narrow perspective by restricting studies to the DCE methodology and focusing solely on 10–24 years olds within the WHO health priority areas taxonomy. In some studies, DCEs absorbed the 10–24 age group and reported combined results based on a single sample of respondents. These studies were excluded from the review. On the other hand, two studies outside the 10–24 age group were included, where most respondents fell in the age group of interest and had a maximum age of 29.4 years. Studies that focused on carers' preferences or had a larger sample of caregivers than young people were also excluded. A comparison of youth versus caregiver preferences felt outside the scope of this review and deserves a standalone assessment.

Included studies had considerable heterogeneity, making it even more challenging to draw firm conclusions. It was impossible to meaningfully synthesise coefficients derived from each study to observe patterns in young people’s choices for several reasons. First, there were an insufficient number of studies with similar aims. Second, for a meaningful comparison, the DCEs studies had to report results in a willingness to pay manner or probability analysis. Since models' coefficients within a study could be interdependent, the simple addition of coefficients could be misleading [22]. In addition, differences in coefficients from separate datasets might be due to scale variance rather than the actual differences [3]. Instead, this review reported the relative importance of each attribute. The relative importance was derived by dividing each attribute coefficient range by the sum of all coefficient ranges within a DCE.

The process of creating, administrating, analysing and reporting DCE is complex and multifaceted. One of the issues with assessing the quality of DCE studies was difficulty assessing all quality criteria. For example, while creating the choice sets, it is a good practice to exclude the dominant set choices as those sets do not usually provide additional information about the relative importance of attributes. However, many studies did not report this step. Having said that, that did not necessarily mean that this step was omitted during the experimental design stage. The missing reporting did not necessarily imply a poor study quality.

The published DCEs literature provided essential insights into the preferences of young people for healthcare interventions and service deliveries. Consideration of adolescent preferences may result in improved acceptance of, engagement in and uptake of interventions, programs and services targeted for this age group. Hence, our findings could be used by clinicians, policymakers, and health care managers to help adapt their practice to young peoples' preferences. To improve the application, we suggest using the minimum quality requirement for reporting and conducting DCEs by using pilot surveys to pre-test the DCE questions, applying the state of art analysis, such as WTP, mixed logit, and conducting the choice validity analysis. Together with the methodological refinements, future research should continue to explore new contexts for the DCE application.

## Supplementary Information

Below is the link to the electronic supplementary material.

Supplementary file1 (DOCX 49 kb)

## Footnotes

1 The first comprehensive systematic literature review of the DCEs in Health Economics to our knowledge was conducted by de Bekker-Grob et al. [15]. Very similar approach was used in 2019 by Soekhai et al. [14]. This study adopted similar indicators to enable comparison to both the [14] and [15].
