# Supplementary material for: The application of discrete choice experiments eliciting young peoples’ preferences for healthcare: a systematic literature review
Source: Eur J Health Econ. 2022 Sep 28;24(6):987–98. doi: 10.1007/s10198-022-01528-9 (PMC10290600; doi:10.1007/s10198-022-01528-9)
Supplement: Supplementary file 1 — Supplementary file1 (DOCX 49 kb) [file 10198_2022_1528_MOESM1_ESM.docx]

The application of discrete choice experiments eliciting young peoples' preferences for healthcare: a systematic literature review

***A supplementary material***

Dr. Galina Williams* [g.williams@cqu.edu.au](mailto:g.williams@cqu.edu.au)

Dr. Irina Kinchin [kinchini@tcd.ie](mailto:Irina.kinchin@uts.edu.au)

*Corresponding author

Health areas included mental health, nutrition, sexual and reproductive health, smoking, unintentional injuries, vaccination and diabetes. Majority of DCEs examined sexual and reproductive health (n=9; 50%) followed by smoking (n14; 22%). One study (*n*=1; 6%) assessed vaccination preferences against severe but rare diseases. The other health areas included mental health (*n=*1; 6%), or preferences for providing information about mood and anxiety disorders, nutrition (*n=*1; 6%), or the role of branding and price in motivating healthier snack consumption. One study related to unintentional injuries (*n=*1; 6%) and, assessed potential demand of young drivers for new modes of public transport designed to prevent risky situations. One study looked at the preferences for clinic related service delivery for type I diabetes (*n*=1, 6%). The studies are described by the WHO [24] target areas below.

### Unintentional injuries (Target area #1) & Alcohol (Target area #5)

A DCE study from Switzerland examined the potential demand of young drivers 14-25 years old (*n*=316) for new modes of public transport designed to reduce and/or prevent the number of risky situations [1]. The study included five attributes, including (1) availability of public transport systems; (2) level of service (frequency); (3) fare; (4) the amount of fine; and, (5) probability of a police checkpoint. Results suggested that young drivers were sensitive to the higher probability of a police checkpoint, more sensitive to a one-month suspension of their driving licence compared to the fine. A higher alcohol attitude has encouraged respondents to switch to new public transport alternatives. They were also more sensitive to “Drinking and Driving Do not Mix” policies.

### Mental health (Target area #2)

One study assessed youth preferences in mental health. This was a study from Canada that assessed preferences for providing information about mood and anxiety disorders [2]. Participants 18-25 years of age (*n*=720; 68% of the selected sample) completed 17 choice tasks presenting combinations of 15 four-level attributes of a mental health information strategy. These attributes related to the content (e.g., self-help strategies); process (e.g., time demands), and the expected outcome (e.g., feeling informed) of a mental health information strategy. The authors analysed and presented results separately for three identified latent classes: virtual participants (28.7%), conventional (30.1%), and low-interest participants (41.2%). The virtual segment preferred an Internet as a source of information for those who experienced anxiety or depression. Conventional participants preferred books or pamphlets recommended by a medical practitioner. The low-interest participants preferred to have short pieces of relevant information All participants preferred to reduce anxiety or depression without psychological approaches or medication. The authors highlighted the need for a combination of active self-help and passive information deliveries through books and Internet channels when developing a mental health information strategy for enhanced utilisation and outcome.

### Sexual and reproductive health (Target area #3)

Nine studies examined preferences of young people for the provision of sexual or reproductive health care initiatives, and their contributing factors, including preferences for human papillomavirus (HPV) vaccination (*n*=3); HIV (*n*=4); integrated service delivery for family planning (FP) and HIV (*n*=1); and specialised FP services delivery (*n*=1).

#### HPV vaccination

Three studies examined preferences associated with HPV vaccination. A Dutch study [3] investigated preferences of 359 secondary school girls aged 12–16 years in order to provide quantitative estimates of the perceived benefits of HPV vaccination and potential vaccine uptake. The study incorporated five attributes, namely: (1) degree of protection against cervical cancer; (2) protection duration; (3) serious side effects (e.g. hospitalisation); (4) mild side effects (e.g. nausea); and (5) age of vaccination. The analysis suggested that young girls made a trade-off between the degree of protection and other vaccine characteristics. Uptake of HPV vaccination may change considerably if girls receive more information about the degree of protection against cervical cancer, the protection duration, and the risk of severe side effects.

Three years later, the same research group conducted a further study into whether and how the girls’ preferences have changed since the start of the HPV vaccination program in Netherlands [4]. The sample comprised 500 secondary school females 11-15 years of age. The analysis applied the same five attributes as the former study. Findings suggested that three years later, the risk of mild side effects and age at vaccination became less important factors. Specifically, most girls preferred vaccination at age 14 years (instead of at age nine years) and a 2-dose scheme (instead of the current 3-dose scheme). The authors recommended for the Dutch national immunisation program not to lower the current target age of 12 years but noted that a 2-dose scheme might result in its higher uptake.

Another DCE study was conducted in 2014 [5]. The study measured preferences for HPV vaccines of 307 adolescents girls aged 13-17 years in the US. This study had different settings and a few different attributes to the Dutch studies [3] and [4], The Dutch studies concentrated on the age of administration in a national vaccination program and did not include genital warts protection or cost attributes. The US study had four attributes including: (1) protection against cervical cancer (50%, 70%, 90%, or 100% (full protection)); (2) protection against genital warts (0% (no protection) or 90%); (3) duration of protection; and, (4) out-of-pocket cost ($0, $100, $300, or $700). Similarly, the Dutch studies’ results suggested that girls might exhibit well-formed preferences over several features of HPV vaccines, such as favouring cervical cancer protection over all other features, and lifetime protection duration. The willingness-to-pay (WTP) for bivalent and quadrivalent HPV vaccines ranged between $400 and $460 in 2008 US$. The additional value of genital warts protection was $145, although cervical cancer efficacy was the most preferred feature. The study also confirmed young females’ preference for a lower dose vaccine (a 1-dose scheme instead of a 3-dose-scheme), which is in line with the finding by the other two studies from the Netherlands.

#### HIV testing

Two studied investigated the preferences for the HIV testing. A study from southern Africa [6] investigated preferences of 341 young people aged 16–25 years in Malawi and Zimbabwe for HIV self-testing (HIVST). The study had six attributes in Malawi and seven attributes in Zimbabwe: (1) test price; (2) sample collection method (Malawi only); (3) type of provider (Malawi only), or provider age and provider residence (Zimbabwe only); (4) location; (5) pre-test support; (6) post-test support (Malawi only); (7) opening hours (Zimbabwe only); and, (8) batched or individual distribution (Zimbabwe only). The study found that young people accepted HIVST, if provided at no or very low cost. Price had a very strong influence on testing choices in both sites, Malawi (U = -4.874, P < 0.01) and Zimbabwe (U = -1.691, P < 0.01). Participants expressed mixed views on oral-fluid tests, weighing perceived benefits with accuracy concerns. There was an expressed lack of trust in health providers and preference for lay community distributors. Participants preferred home-based distribution as a more convenient delivery mode. Issues of autonomy, control, respect and confidentiality emerged as key qualitative themes. Overall, HIVST addressed youth-specific barriers to standard HIV testing.

The second study looked at the preferences for HIV testing among South African youth [7]. Each respondent had to answer 18 questions with two unlabeled options. Each option contained 6 attributes: (1) pre-testing information (social media, SMS, pamphlet); (2) incentive amount (zero, R25, R50); (3) incentives type (food voucher, data bundle, cash); (4) testing support (alone, with a friend, with parents); (5) location (clinic, home, school) and, (6) type (oral swab, finger prick). The results showed that youth preferred to go for a test alone, to receive HIV-related information by SMS and to receive a food voucher as an incentive to do the test. The respondents preferred to do the test in clinic or at home with the preferred testing method being a finger prick.

#### Integrated family planning (FP), HIV services and vaccination

A study from rural Malawi examined 537 young people’s (15-24 years old) preferences for integrated family planning (FP) and HIV services to improve the acceptability and uptake of such services [8]. The analysis contained six attributes: (1) age and (2) gender of service provider; (3) confidentiality; (4) availability of HIV services; (5) special youth friendly service components; and, (6) price. Results showed that young people valued confidentiality and the availability of HIV services, including HIV counselling and testing (HCT) and HIV treatment. Results showed that the most preferred service package included confidentiality of services, HCT and HIV treatment, and recitation and sport activities. Estimates of WTP indicated that respondents were willing to pay up to USD$1.76 for confidentiality, USD$0.65 for a service offering both HCT and HIV treatment and USD$0.26 for service including sports activities.

Two studies on various aspects of HIV have been conducted in South Africa. A study on vaccination against HIV was conducted by Minnis et al [9]. The study investigated the youth’s preferences using five attributes: (1) the product form (injection or implant); (2) duration; (3) place of procedure; (4) delivery location on the body; and (5) the level of pain. While the WTP/WTA were not estimated, the results showed the importance of fewer dosage frequency and the injection product form.

The second study by Galarraga et al [10] examined youth’s preferences for the programs that help young people to take their medicine every day. The DCE used unlabeled alternatives with an opt-out option. Profiles had five attributes: (1) incentive amount; (2) incentive format; (3) who receives an incentive; (4) who can participate; and (5) incentive delivery mode. The results showed that programs with higher levels of incentives and in cash were preferred by respondents to the food or fashion vouchers. Respondents were willing to forgo US$9 from the maximum incentive amount (US$ 115) in order for the incentive to be in cash rather than in a form of a voucher. The preferences were to have a mixed group of participants (value of US$19.6) and in the clinic (US$6.5).

#### Family planning (FP)

Another DCE study from rural Malawi quantified the impact of service provider characteristics on young people’s (aged 15-24; *n*=540) choice of FP service provider [11]. The analysis considered six attributes: (1) the distance between participants’ home and the service delivery point; (2) frequency of service delivery; (3) waiting time at the facility; (4) service providers’ attitude; (5) availability of FP commodities; and, (6) price. The results were presented in terms of odds ratios (OR), whereby an OR >1 for an attribute suggested that as the level of the attribute increased, option one was preferred over option 2. Service provider attitude (OR=2.45 for the government service, 1.99 for the private service and 1.88 for the community-based service) and FP commodities (OR=2.48 for the government service, 2.33 for the private service and 3.85 for the community-based service) were among the strongest factors influences the choice of service providers. The results showed that youth preferred to have community-based services to facility-based services. Similarly to the finding by [6] and [8], respondents expressed a strong preference for a lower price option.

A DCE study was conducted in Australia that assessed vaccination preferences in 695 adolescents aged 15-19 years [12]. Six attributes were assessed including (1) disease severity; (2) target for protection; (3) price; (4) location of vaccination provision; (5) potential side effects; and, (6) vaccine delivery method. The results showed that the stronger preferences were for: vaccination in the case of a life-threatening illness (p<0.001), lower price vaccinations (p<0.001), mild but common side effects (p = 0.004), delivery via a skin patch (p<0.001) and is administered by a family practitioner (p<0.001). WTP for a vaccine targeting a life-threatening illness than a mild-moderate illness was AU$394.28 (95%CI: AU$348.40 to AU$446.92) , for vaccination at a family practitioner clinic than a council immunisation clinic AU$37.94 (95%CI: AU$19.22 to AU$57.39) , for common but mild and resolving side effects compared to rare but severe side effects AU$23.01 (95%CI: AU$7.12 to AU$39.24) , and for delivery via a skin patch than injection AU$51.80 (95%CI: AU $30.42 to AU$73.70)..

### Nutrition (Target area #4)

An USA study investigated the role of branding and price in motivating American children aged 8-11 (*n*=116) to choose healthier snack options [13]. The analysis included three attributes: (1) product type; (2) brand; and, (3) price. The authors found a child’s liking of the brand determines a child’s motivation to choose a product. The extent of children’s experience with money influenced their price responsiveness in those children who received an allowance were primarily the ones buying food snacks. Hence, higher prices for energy-dense, nutrient-poor snacks could be successful in motivating children to choose a healthier option.

A DCE study from Ireland examined the potential demand of young adults (25+-4.4 years; *n*=24) for clinic related service delivery [14]. They used the following eight attributes: (1) waiting time; (2) healthcare professional; (3) choice of healthcare professional seen; (4) optional services; (5) flexible booking system; (6) blood glucose diary; (7) HbA_1c_ test and (8) cost. The results from conditional logit analysis suggested that respondents preferred shorter waiting time, to see nurse and consultant selected by the respondent, flexible booking system and at a low price. Respondents did not have a preference to see a nurse and a doctor, relative to a nurse alone, or other optional services (e.g. seeing dietitians or psychologists), type of HbA_1c_ test and digital blood glucose diaries over paper-based diaries. Respondents were willing to pay €0.95 to reduce the waiting time by 1 minute. They valued visiting a consultant at €41.53 per year relative to visiting the nurse only. Flexible booking system with easy to cancel and reschedule attracted €12 per year premium.

### Smoking cessation (Target area #6)

A study from Canada by [15] surveyed young females aged 16-24 years old (*n*=448). This study considered five attributes: (1) pack structure (slim, lipstick, booklet, traditional); (2) brand (“Vogue,” “du Maurier”); (3) branding (branded, plain); (4) warning label size (50%, 75%); and, (5) price ($8.45, $10.45). Pack structure found to be the most influential factor for choosing cigarettes as it was strongly correlated with the taste related perceptions. Price and branding were less significant, while warning label size did not significantly impact perceptions of taste.

Another DCE study examined attributes of a smoking cessation program using a sample of university students (*n*=191; 23 years old with standard deviation of 6.0) from Lebanon [16]. The analysis contained five attributes: (1) medication type; (2) availability of support; (3) risk of side effects; (4) distance travelled; and, (5) price. Results showed that students were less interested in the pill form of smoking cessation treatment, as reported by smokers in other studies with general populations. Participants were also willing to make trade-offs to be smoke-free. On average, smokers were willing to pay US$ 69 for cessation support. Respondents were willing to give up US$ 70 to avoid an additional 10% risk of minor side effects and US$ 12 to avoid an addition kilometre of travel to the nearest pharmacy. Heavy smokers were the least responsive group and had the lowest demand elasticities.

A DCE study from the USA estimated young adults’ (aged 18-22 years old; n=2,003) preferences for cigarettes and e-cigarettes [17]. The study considered the following four attributes: (1) flavours, (2) short-term health risks to self, (3) second-hand smoke risks and (4) price. The results presented in the form of the basic MNL model and the latent class model. The latent class model uncovered two groups of respondents: “prefer smoking group” and “prefer vaping group”. The first group (37% of the sample) had strong preferences for cigarettes, while the second group (63% of the sample) had stronger preferences for reusable e-cigarettes. The results showed that if the “prefer vaping group” switched from e-cigarettes, they would be most likely to abstain from all products. The “prefer smoking group” was more price-sensitive than the “prefer vaping group”.

An Eastern Mediterranean study looked at the demand for waterpipe smoking sessions (18-29 years old; n=1,859) [18]. The choices included three within-subjects attributes: (1) flavours, (2) nicotine content, and (3) prices as well as one between-subjects attribute: (1) presence of health warning. MNL showed that all attributes were statistically significant with flavour attribute accounting for 81.4% of choice, while the presence of health warning was not statistically significant.

### References

1. Scagnolari, S., J. Walker, and R. Maggi, *Young drivers' night-time mobility preferences and attitude toward alcohol consumption: A Hybrid Choice Model.* Accident; analysis and prevention, 2015. **83**: p. 74-89.

2. Cunningham, C.E., et al., *Modeling mental health information preferences during the early adult years: A discrete choice conjoint experiment.* Journal of Health Communication, 2014. **19**(4): p. 413-440.

3. de Bekker-Grob, E.W., et al., *Girls' preferences for HPV vaccination: A discrete choice experiment.* Vaccine, 2010. **28**(41): p. 6692-6697.

4. Hofman, R., et al., *Have preferences of girls changed almost 3 years after the much debated start of the HPV vaccination program in the Netherlands? A discrete choice experiment.* PLoS ONE, 2014. **9**(8).

5. Brown, D.S., et al., *Adolescent girls' preferences for HPV vaccines: A discrete choice experiment*, in *Advances in Health Economics and Health Services Research*. 2014. p. 93-121.

6. Indravudh, P.P., et al., *'I will choose when to test, where i want to test': Investigating young people's preferences for HIV self-testing in Malawi and Zimbabwe.* AIDS, 2017. **31**: p. S203-S212.

7. Chetty-Makkan, C.M., et al., *Youth Preferences for HIV Testing in South Africa: Findings from the Youth Action for Health (YA4H) Study Using a Discrete Choice Experiment.* AIDS and Behavior, 2021. **25**(1): p. 182-190.

8. Michaels-Igbokwe, C., et al., *Designing a package of sexual and reproductive health and HIV outreach services to meet the heterogeneous preferences of young people in Malawi: results from a discrete choice experiment.* Health Economics Review, 2015. **5**(1).

9. Minnis, A.M., et al., *Preferences for long-acting Pre-Exposure Prophylaxis (PrEP) for HIV prevention among South African youth: results of a discrete choice experiment.* Journal of the International Aids Society, 2020. **23**(6): p. 10.

10. Galárraga, O., et al., *iSAY (incentives for South African youth): Stated preferences of young people living with HIV.* Social Science & Medicine, 2020. **265**.

11. Michaels-Igbokwe, C., et al., *Young People's Preferences for Family Planning Service Providers in Rural Malawi: A Discrete Choice Experiment.* PLoS ONE, 2015. **10**(12).

12. Wang, B., et al., *Adolescent values for immunisation programs in Australia: A discrete choice experiment.* PLoS ONE, 2017. **12**(7).

13. Hartmann, M., et al., *Children's purchase behavior in the snack market: Can branding or lower prices motivate healthier choices?* Appetite, 2017. **117**: p. 247-254.

14. Mc Morrow, L., et al., *The preferences of young adults with Type 1 diabetes at clinics using a discrete choice experiment approach: the D1 Now Study.* Diabetic medicine : a journal of the British Diabetic Association, 2018. **35**(12): p. 1686-1692.

15. Kotnowski, K., et al., *The impact of cigarette packaging design among young females in canada: Findings from a discrete choice experiment.* Nicotine and Tobacco Research, 2016. **18**(5): p. 1348-1356.

16. Salloum, R.G., et al., *Assessing preferences for a university-based smoking cessation program in Lebanon: A discrete choice experiment.* Nicotine and Tobacco Research, 2015. **17**(5): p. 580-585.

17. Buckell, J. and J.L. Sindelar, *The impact of flavors, health risks, secondhand smoke and prices on young adults' cigarette and e-cigarette choices: a discrete choice experiment.* Addiction (Abingdon, England), 2019. **114**(8): p. 1427-1435.

18. Salloum, R.G., et al., *Individual-level determinants of waterpipe smoking demand in four Eastern-Mediterranean countries.* Health promotion international, 2018.

**Data extracted**

| Year study |
| --- |
| Year published |
| N (done) |
| Lead Author (Year) |
| Country |
| Health area (disease) |
| Main Study objectives |
| Samples compared |
| Attributes types (qualitative, quantitative) |
| Price/Cost attribute? 1=yes, 0=no |
| Attributes Tested |
| N of attributes |
| Piloting |
| Framing of choice tasks |
| Generated in all groups? |
| Choice; Outcomes |
| Design type; Full factorial, fractional, not reported |
| Design plan: main effects only, main effect and selected 2 way interactions, full factorial, na |
| Design source: software package, expert, not reported |
| Method to create choice sets: Single profiles, random, foldover, d-efficiency, not reported |
| Number of choices per respondent |
| Administration of survey |
| Reminders |
| Response rate |
| Perception of ease of tasks |
| Completion time |
| Choice same in all groups? |
| Analysis |
| Validity |
| Results |
